# The Proapoptotic Action of Pyrrolidinedione–Thiazolidinone Hybrids towards Human Breast Carcinoma Cells Does Not Depend on Their Genotype

**DOI:** 10.3390/cancers16162924

**Published:** 2024-08-22

**Authors:** Nataliya Finiuk, Yuliia Kozak, Agnieszka Gornowicz, Robert Czarnomysy, Marlena Tynecka, Serhii Holota, Marcin Moniuszko, Rostyslav Stoika, Roman Lesyk, Krzysztof Bielawski, Anna Bielawska

**Affiliations:** 1 Department of Regulation of Cell Proliferation and Apoptosis, Institute of Cell Biology of National Academy of Sciences of Ukraine, Drahomanov 14/16, 79005 Lviv, Ukraine; juliana.kozzak@gmail.com (Y.K.); stoika.rostyslav@gmail.com (R.S.); 2 Department of Biotechnology, Faculty of Pharmacy, Medical University of Bialystok, Kilinskiego 1, 15-089 Białystok, Poland; agnieszka.gornowicz@umb.edu.pl (A.G.); anna.bielawska@umb.edu.pl (A.B.); 3 Department of Synthesis and Technology of Drugs, Faculty of Pharmacy, Medical University of Bialystok, Kilinskiego 1, 15-089 Białystok, Poland; robert.czarnomysy@umb.edu.pl (R.C.); krzysztof.bielawski@umb.edu.pl (K.B.); 4 Centre of Regenerative Medicine, Medical University of Bialystok, Kilinskiego 1, 15-089 Białystok, Poland; marlena.tynecka@umb.edu.pl (M.T.); marcin.moniuszko@umb.edu.pl (M.M.); 5 Department of Pharmaceutical, Organic and Bioorganic Chemistry, Danylo Halytsky Lviv National Medical University, Pekarska 69, 79010 Lviv, Ukraine; golota_serg@yahoo.com (S.H.); dr_r_lesyk@org.lviv.net (R.L.); 6 Department of Biotechnology and Cell Biology, Medical College, University of Information Technology and Management in Rzeszów, Sucharskiego 2, 35-225 Rzeszów, Poland

**Keywords:** hybrid molecules, pyrrolidinedione–thiazolidinone, breast carcinoma cells, ER, PR, HER2 genotypes, toxicity, apoptosis, autophagy

## Abstract

**Simple Summary:**

Breast cancer is one of the most frequent tumors worldwide, based on the number of new cases and deaths. Unfortunately, the low selectivity of action and the rapid development of multiple drug resistances remain the main disadvantages of anticancer compounds. The search for new agents with pronounced antitumor activity is an urgent task in modern biology and medicine. We focused on the investigation of the antitumor potential of novel hybrid pyrrolidinedione–thiazolidinone derivatives. The synthesized derivatives are effective and selective agents that exhibit their antitumor effects in breast carcinoma cells via (1) inhibiting viability, proliferation, and the ability to form colonies; (2) inducing extrinsic and intrinsic apoptotic pathways; and (3) decreasing the level of proteins associated with autophagy, invasion, and metastasis. Our results indicate that synthesized derivatives are potential candidates for deeper exploration of their therapeutic efficiency.

**Abstract:**

The development of new, effective agents for the treatment of breast cancer remains a high-priority task in oncology. A strategy of treatment for this pathology depends significantly on the genotype and phenotype of human breast cancer cells. We aimed to investigate the antitumor activity of new pyrrolidinedione–thiazolidinone hybrid molecules **Les-6287**, **Les-6294**, and **Les-6328** towards different types of human breast cancer cells of MDA-MB-231, MCF-7, T-47D, and HCC1954 lines and murine breast cancer 4T1 cells by using the MTT, clonogenic and [^3^H]-Thymidine incorporation assays, flow cytometry, ELISA, and qPCR. The studied hybrids possessed toxicity towards the mentioned tumor cells, with the IC_50_ ranging from 1.37 to 21.85 µM. Simultaneously, these derivatives showed low toxicity towards the pseudonormal human breast epithelial cells of the MCF-10A line (IC_50_ > 93.01 µM). **Les-6287** at 1 µM fully inhibited the formation of colonies of the MCF-7, MDA-MB-231, and HCC1954 cells, while **Les-6294** and **Les-6328** did that at 2.5 and 5 µM, respectively. **Les-6287** suppressed DNA biosynthesis in the MCF-7, MDA-MB-231, and HCC1954 cells. At the same time, such an effect on the MCF-10A cells was significantly lower. **Les-6287** induces apoptosis using extrinsic and intrinsic pathways via a decrease in the mitochondrial membrane potential, increasing the activity of caspases 3/7, 8, 9, and 10 in all immunohistochemically different human breast cancer cells. **Les-6287** decreased the concentration of the metastasis- and invasion-related proteins MMP-2, MMP-9, and ICAM-1. It did not induce autophagy in treated cells. In conclusion, the results of our study suggest that the synthesized hybrid pyrrolidinedione–thiazolidinones might be promising agents for treating breast tumors of different types.

## 1. Introduction

Cancer ranks as the second-leading cause of death [1]. Despite advancements in cancer treatment approaches (immune-, hormone-, cytokine-, and RNA-based therapies), chemotherapy is still an important therapeutic option [2,3]. However, its use is often limited by significant side effects, systemic toxicity, and drug resistance [4,5].

Breast cancer is the major cause of morbidity and mortality among other malignant tumors in the world. This oncological disease is one of the serious public health challenges worldwide that kills more European women than any other type of cancer [1,6]. Breast cancer can be classified into four molecular subgroups according to how the estrogen receptor (ER), the progesterone receptor (PR), and the human epidermal growth factor receptor 2 (HER2) are expressed: luminal A (ER+ and/or PR+, and HER2−), luminal B (ER+ and/or PR+, and HER2+), HER2+ (ER−, PR−, and HER2+), and triple-negative (TNBC: ER−, PR−, and HER2−) [7].

Immunohistochemical determination of breast cancer subtypes, in addition to their diagnostic and prognostic value, plays a crucial role in choosing the most effective treatment strategy [8]. In recent years, targeted therapy has been used for breast cancer treatment, which includes HER2-targeted therapy (trastuzumab, pertuzumab), ER/PR-targeted therapy (tamoxifen, letrozole, exemestane), as well as tyrosine kinase, PARP, CDK4/6, and mTOR inhibitors (lapatinib, neratinib, ribociclib, tucatinib, and others) [9,10,11,12]. Unfortunately, the rapid development of drug resistance is the main problem with modern targeted drugs, which leads to the rapid progression of cancer [13]. Moreover, TNBC does not respond to traditional treatments, and most targeted drugs are useless. Therefore, chemotherapy remains one of the main systematic methods of breast cancer treatment, but its effectiveness in many cases is low. For enhancing effectiveness, chemotherapy drugs can be used in combination with other therapeutic approaches, such as targeted immunotherapy [14]. In addition, the novel, highly effective compound can be used for the synthesis of antibody–drug conjugates. With the approval of trastuzumab emtansine and trastuzumab deruxtecan, this approach is the focus of world scientists [15]. So, the development of new, highly effective chemotherapeutic agents for the treatment of immunohistochemically different subtypes of breast cancer is an acute task of modern pharmacology and medicine.

**Figure 1:**
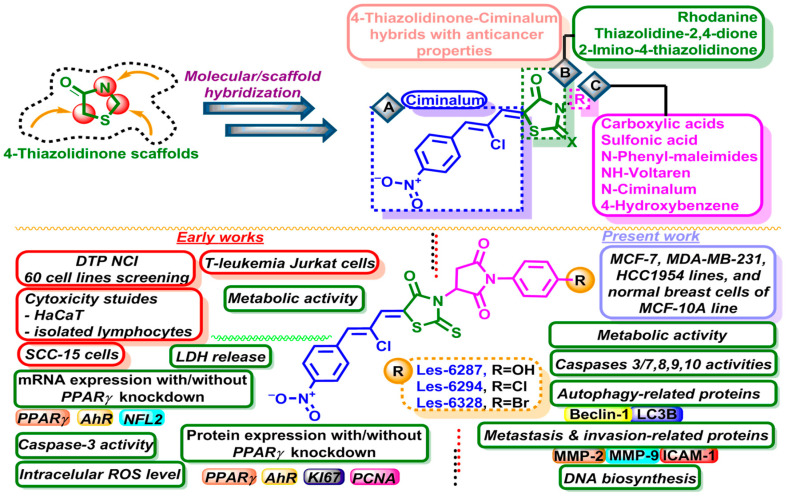
Background and design of the present studies. Structures of the studied hybrids **Les-6287**, **Les-6294**, and **Les-6328**.

Applying the pharmacophore/molecular hybridization approaches has proven to be an effective strategy for designing and searching for anticancer-hit compounds with 4-thiazolidinone scaffolds [16,17,18,19]. Ciminalum and pyrrolidinedione-containing molecules possess a privileged place in the design of different chemotypes of 4-thiazolidinone hybrids with anticancer activity (Figure 1). So, a series of pyrrolidinedione–thiazolidinone hybrid molecules with potential anticancer properties have been reported [20,21,22,23,24]. Buzun et al. described a Ciminalum-4-thiazolidinone hybrid with an impact on leukemia, melanoma, colon, glioma, gastric, and breast tumor NCI cell lines with a GI_50_ value of 1.57 μM [21]. The same authors also reported the cytotoxic effect of one more Ciminalum-4-thiazolidinone hybrid on breast carcinoma MCF-7 and MDA-MB-231 cells with IC_50_ values of 5–15 μM at 24 h. It induced mitochondria-dependent apoptosis and decreased the topoisomerase II concentration [22]. Finiuk et al. described a series of the Ciminalum-bearing pyrrolidinedione-4-thiazolidinone hybrid molecules **Les-6294**, **Les-6287**, and **Les-6328** (Figure 1) that inhibited the metabolic activity of Jurkat, KB3-1, HeLa, HCT116, A549, and U251 cells with the IC_50_ values of 1–9 µM following 72 h of incubation [23,24].

With the aim of in-depth studies and as a part of our ongoing systematic research of the anticancer properties of Ciminalum–pyrrolidinedione–4-thiazolidinone hybrids, herein we report on the evaluation of the impact of the hybrid molecules **Les-6287**, **Les-6294**, and **Les-6328** (Figure 1) [23,24] on the viability, metabolic activity, DNA synthesis, apoptosis, and autophagy induction in breast carcinoma cells.

## 2. Materials and Methods

### 2.1. Materials

All chemicals and solvents were purchased from commercial suppliers. The 3-(4,5-dimethylthiazol-2-yl)-2,5-diphenyltetrazolium bromide (MTT, 98%, Product No. M2128), dimethyl sulfoxide (DMSO, ReagentPlus^®^, ≥99.5%, Product No. D5879), β-mercaptoethanol (Product No. M3148), methanol (≥99.9%, Product No. 34860), crystal violet (Product No. C0775), sodium hydroxide (Product No. 484024), sodium dodecyl sulfate (SDS, ≥98.5%, Product No. L3771), sodium chloride (ReagentPlus^®^, ≥99%, Product No. S9625), doxorubicin (Product No. PHR1789), and Tris (2-amino-2-(hydroxymethyl)-1,3-propanediol, ≥99.8%, Product No. 93362) were purchased from Sigma-Aldrich (St. Louis, MO, USA). Ethanol (96%) was obtained from Avantor Performance Materials (Gliwice, Poland). The trichloroacetic acid (TCA, pure p.a. ≥ 98%, Cat. No. 76-03-09) was from Chempur (Piekary Slaskie, Poland). The human breast carcinoma MCF-7 (HTB-22), MDA-MB-231 (HTB-26), T-47D (HTB-133), and HCC1954 (CRL-2338) cells, normal human breast epithelial MCF-10A (CRL-10317) cells, and murine breast cancer 4T1 (CRL-2539) cells were provided by the American Type Culture Collection (ATCC, Manassas, VA, USA). Dulbecco’s Minimal Eagle Medium (DMEM, Cat. No. 11995065), RPMI-1640 medium (Cat. No. 11875093), fetal bovine serum (FBS, Cat. No. 10091148), horse serum (Cat. No. 16050122), phosphate-buffered saline (PBS, Cat. No. J61196.AP), 0.05% trypsin with 0.02% EDTA (Cat. No. 25300054), glutamine (Cat. No. 25030081), and penicillin/streptomycin solution (Cat. No. 15140122) were from Gibco (San Diego, CA, USA). An MEGM Mammary Epithelial Cell Growth Medium BulletKit (Product No. CC-3150) was from Lonza Bioscience (Basel, Switzerland). [^3^H]-thymidine (7 Ci/mmol, Cat. No. MT6031) was received from Moravek Biochemicals (Brea, CA, USA), and Scintillation Cocktail Ultima Gold XR (Product No. 6013119) was received from PerkinElmer (Waltham, MA, USA). DNase-free RNase A Solution (Cat. No. M6101) was from Promega (Madison, WI, USA). FITC Annexin V Apoptosis Detection Kit II (Cat. No. 556547) and JC-1 MitoScreen Kit (Cat. No. 551302) were purchased from BD Pharmingen (San Diego, CA, USA). The propidium iodide (Cat. No. 638), FAM-FLICA^®^ Caspase-3/7 Assay Kit (Cat. No. 93), FAM-FLICA^®^ Caspase-8 Assay Kit (Cat. No. 99), FAM-FLICA^®^ Caspase-9 Assay Kit (Cat. No. 912), and FAM-FLICA^®^ Caspase-10 Assay Kit (Cat. No. 922) were purchased from ImmunoChemistry Technologies (Bloomington, MN, USA). High-sensitivity human Beclin-1 SimpleStep ELISA Kit (Cat. No. ab254511), and high-sensitivity human SimpleStep ELISA Kits for MMP-2 (Cat. No. ab267813), MMP-9 (Cat. No. ab246539), and ICAM-1 (CD54, Cat. No. ab174445) were from Abcam (Cambridge, UK); LC3B ELISA Kit (Cat. No. E4774Hu) was from BT LAB (Shanghai, China). RLT buffer (Cat. No. 79216) and the RNeasy Mini Kit (Cat. No. 74104) were from Qiagen (Wrocław, Poland). The High-Capacity cDNA Reverse Transcription Kit (Cat. No. 4368814) was purchased from Thermo Fisher Scientific Inc., Waltham, MA, USA.

### 2.2. Studied Compounds

The synthesis and characterization of pyrrolidinedione–thiazolidinone hybrid molecules **Les-6287** (1-(4-hydroxyphenyl)-3-[5-[2-chloro-3-(4-nitrophenyl)prop-2-enylidene]-4-oxo-2-thioxothiazolidine-3-yl]pyrrolidine-2,5-dione) and **Les-6294** (1-(4-chlorophenyl)-3-[5-[2-chloro-3-(4-nitrophenyl)prop-2-enylidene]-4-oxo-2-thioxothiazolidine-3-yl]pyrrolidine-2,5-dione) was reported in [23] and **Les-6328** (1-(4-bromophenyl)-3-[5-[2-chloro-3-(4-nitrophenyl)prop-2-enylidene]-4-oxo-2-thioxothiazolidine-3-yl]pyrrolidine-2,5-dione)—in [24]. The 100 mM stock solutions of pyrrolidinedione–thiazolidinones were prepared in DMSO.

### 2.3. Culture of MCF-7, MDA-MB-231, HCC1954, T-47D, 4T1, and MCF-10A Cells

The MCF-7 and MDA-MB-231 cells were cultured in the DMEM medium containing 10% of the FBS, HCC1954, T-47D, and 4T1 cells in the RPMI-1640 medium with the addition of 10% of the FBS, and MCF-10A cells in MEGM Mammary Epithelial Cell Growth Medium BulletKit supplemented with penicillin/streptomycin cocktail and 5% of horse serum. The cells were cultured in a humanized atmosphere at 5% CO_2_ and 37 °C. After trypsinization, cells were counted using a Scepter 3.0 handheld automated cell counter (Millipore, Burlington, MA, USA) [25].

### 2.4. The MTT Assay

The MTT test was used to assess the metabolic activity of cells treated with the investigated substances following the Sigma-Aldrich manufacturer’s instructions [23]. Briefly, compounds were added in 100 µL of the cultural medium, and cells were incubated for the next 24 and 48 h. Doxorubicin was used as a reference drug. The DMSO was added to dissolve the crystals of formazan. We used the ThermoScientific Evolution 201 UV–VIS spectrophotometer (ThermoFisher Scientific, Waltham, MA, USA) to measure the absorbance of formazan at 570 nm. The initial value of the relative number of cells in control (under 0.1% of the DMSO) was accepted as 100%. We used GraphPad Prism Version 9 to calculate the half-maximal inhibition concentration (IC_50_) of the studied compounds (the concentration of the drug that reduced cell viability by 50%).

### 2.5. The Clonogenic Assay

The clonogenic assay was carried out utilizing the previously published methodology [26]. The MCF-7, MDA-MB-231, HCC1954, and MCF-10A cells (500 cells/2 mL) were seeded in a 12-well plate overnight. Afterward, studied derivatives or doxorubicin (1, 2.5, 5, 10, and 50 µM) were added to cells. The media was changed after 72 h to a compound-free one, and the cells were cultured for the following 14 days. Then, the cells were washed, fixed, and stained with 0.1% crystal violet. After washing, the plates were dried at room temperature. The colonies were recorded by ImageJ software (Version 1.30). The value of colonies in control (non-treated cells) was accepted as 100%. The effect of compounds on the colony-forming ability of cells was expressed as the percentage of colonies compared to the control.

### 2.6. [^3^H]-Thymidine Incorporation Assay

Following a 24 h cell treatment, the incorporation of [^3^H]-thymidine into the DNA of the breast tumor and normal cells was used to measure the antiproliferative activity of **Les-6287**. The MCF-7, MDA-MB-231, HCC1954, and MCF-10A cells were seeded into six-well plates (density 3 × 10^5^ cells/well) and cultured for 24 h. Then, they were incubated under the same conditions for the next 24 h with a growth medium containing the tested compounds and reference drug (doxorubicin) at different concentrations (1, 2.5, 5, 10, 50 µM). After 24 h, the medium was changed to a fresh one without FBS. A total of 0.5 µCi of tritium-labeled thymidine (specific activity: 7 Ci/mmol) was added to cells for 4 h. The cells were washed with 1 mL of 0.05 M Tris-HCl buffer pH 7.4 containing 0.11 M NaCl and 1 mL of 5% trichloroacetic acid (TCA). Each well was filled with 1 mL of a 0.1 M NaOH solution containing 1% SDS to complete cell lysis. After 5 min, the acquired cell lysates were put into scintillation vials and pre-filled with two milliliters of scintillation liquid. The Scintillation Counter 1900 TR, TRI-CARB (Packard, Perkin Elmer, Inc., San Jose, CA, USA) was used to record the results [25]. The effect of compounds on DNA synthesis was expressed as % of the control (non-treated cells).

### 2.7. Flow Cytometry of Apoptosis Induction in Breast Tumor Cells

The FITC Annexin V Apoptosis Detection Kit II and a flow cytometer (BD FACSCanto II, BD Biosciences Systems, San Jose, CA, USA) were used to detect the effect of studied derivatives on apoptosis induction in MCF-7 and MDA-MB-231 lines incubated for 24 h with **Les-6287** (1 and 1.5 µM), reference drug–doxorubicin (1 µM), and 0.15% of solvent DMSO (dose that corresponds to its content at 1.5 µM of compound **Les-6287**). The assay was performed following the manufacturer’s instructions and the previously described methodology [25]. Cells were washed twice with cold PBS and resuspended in the Binding Buffer from the Kit. The FITC Annexin V and propidium iodide (PI) were added to 100 µL of cell suspension and incubated for 15 min at room temperature, protected from light. Then, 300 µL of Binding Buffer was added, and samples were analyzed using a flow cytometer. A total of 10,000 events were measured. The FACSDiva software (Version 6.1.3, BD Biosciences Systems, San Jose, CA, USA) was applied to analyze all flow cytometry data. The BD Cytometer Setup and Tracking Beads (BD Biosciences, San Diego, CA, USA) were used for the calibration of the flow cytometer [25].

### 2.8. Assessment of Changes in Mitochondrial Membrane Potential

The JC-1 MitoScreen kit (BD Pharmigen, San Diego, CA, USA) and a flow cytometer (BD FACSCanto II) were used to detect the decrease in mitochondrial membrane potential (MMP, ΔΨm) under the action of the studied compounds. The assay was performed following the manufacturer’s instructions and the previously described methodology [25,27,28]. The MCF-7 and MDA-MB-231 cells were treated for 24 h with **Les-6287** (1 and 1.5 µM), reference drug–doxorubicin (1 µM), and 0.15% of solvent DMSO (dose that corresponds to its content at 1.5 µM of compound **Les-6287**). Following washing, the cells were further suspended in 0.5 mL of buffer with 10 µg/mL JC-1 and incubated for 15 min at room temperature in the dark. Then, cells were washed twice with buffer, resuspended in 300 µL PBS, and analyzed [25].

### 2.9. Caspases 3/7, 8, 9, and 10 Enzymatic Activity Assays

The assay was performed following the manufacturer’s instructions and the previously described methodology [25]. The MCF-7 and MDA-MB-231 cells were treated for 24 h with **Les-6287** (1 and 1.5 µM), reference drug–doxorubicin (1 µM), and 0.15% of solvent DMSO (dose that corresponds to its content at 1.5 µM of compound **Les-6287**). The cells were gathered, rinsed twice with chilled PBS, and reconstituted in an Apoptosis Wash Buffer. Then, 10 µL of freshly diluted FLICA solution was added to 290 µL of cell suspension and incubated for an hour at 37 °C. Following two rounds of washing with 2 mL of Apoptosis Wash Buffer, the cells were centrifuged, resuspended in 300 µL of the buffer, and then analyzed [25].

### 2.10. RNA Isolation and Quantitative PCR (qPCR)

The MCF-7 and MDA-MB-231 cells were treated for 24 h with **Les-6287** and reference drug–doxorubicin (both at 1 µM concentration), harvested, and lysed in RLT buffer (Qiagen, Venlo, The Netherlands) supplemented with 1% of β-mercaptoethanol. Total RNA was isolated using the RNeasy Mini Kit (Qiagen, Venlo, The Netherlands) according to the manufacturer’s protocol and quantified on a NanoDrop spectrophotometer (NanoDrop™ One/OneC, ThermoFisher, Waltham, MA, USA). Next, 1 μg of RNA from every sample was reverse transcribed using a High-Capacity cDNA Reverse Transcription Kit (ThermoFisher, Waltham, MA, USA) according to the standard manufacturer’s instructions. The expression of genes, namely *MAP1LC3b* (Bio-Rad, Hercules, CA, USA) and *BECN1* (Bio-Rad, Hercules, CA, USA), was evaluated using SsoAdvanced Universal SYBR Green Supermix (Bio-Rad, Hercules, CA, USA) on the StepOne Plus system (ThermoFisher, Waltham, MA, USA). StepOne Software v2.3 (Thermo Fisher Scientific, Waltham, MA, USA) was used for results calculations. The results were normalized to *GAPDH* (Bio-Rad, Hercules, CA, USA) and untreated cells (control). The primers used to investigate the expression of the mentioned genes are listed in Table 1. Data were presented as a relative expression using the 2^−ΔΔCt^ method.

### 2.11. ELISA Measurement of Beclin-1, LC3B, MMP-2, MMP-9, and ICAM-1

The MCF-7 and MDA-MB-231 cells were treated for 24 h with **Les-6287** (1, 1.5, and 2 μM), as well as a reference drug–doxorubicin (1, 1.5, and 2 μM). In order to determine the Beclin-1 concentration in the cell lysates, the SimpleStep ELISA (Abcam, Cambridge, UK) Kit was used. MMP-2, MMP-9, and ICAM-1 concentrations were measured in culture supernatants using high-sensitivity human SimpleStep ELISA kits (Abcam, Cambridge, UK) according to the manufacturer’s protocols. The LC3B ELISA kit (BT LAB, Shanghai, China) was used to check the protein concentration in cell lysates from analyzed breast cancer cells. The procedure for preparing the samples was shown previously [29].

### 2.12. Statistical Analysis

Results were presented as mean ± standard deviation (M ± SD). Data analysis of gene expression studies was performed with GraphPad Prism Version 10 software; data from ELISA’s measurements were analyzed with GraphPad Prism Version 6.0; and other results were analyzed and illustrated with GraphPad Prism Version 9 (GraphPad Software, San Diego, CA, USA). The differences between the control and tested groups were evaluated by means of ANOVA tests (*p* < 0.05).

## 3. Results

### 3.1. Cytotoxicity of Derivatives towards Breast Cancer Cells

Continuing screening of the antitumor activity of pyrrolidinedione–thiazolidinone hybrid molecules, we studied in vitro cytotoxic activities towards human breast carcinoma MCF-7, T-47D, MDA-MB-231, and HCC1954 cells, murine breast carcinoma 4T1 cells, and normal human breast epithelial MCF-10A cells using the MTT test, assays on the incorporation of [^3^H]-thymidine into DNA, and clonogenic assays.

**Figure 2:**
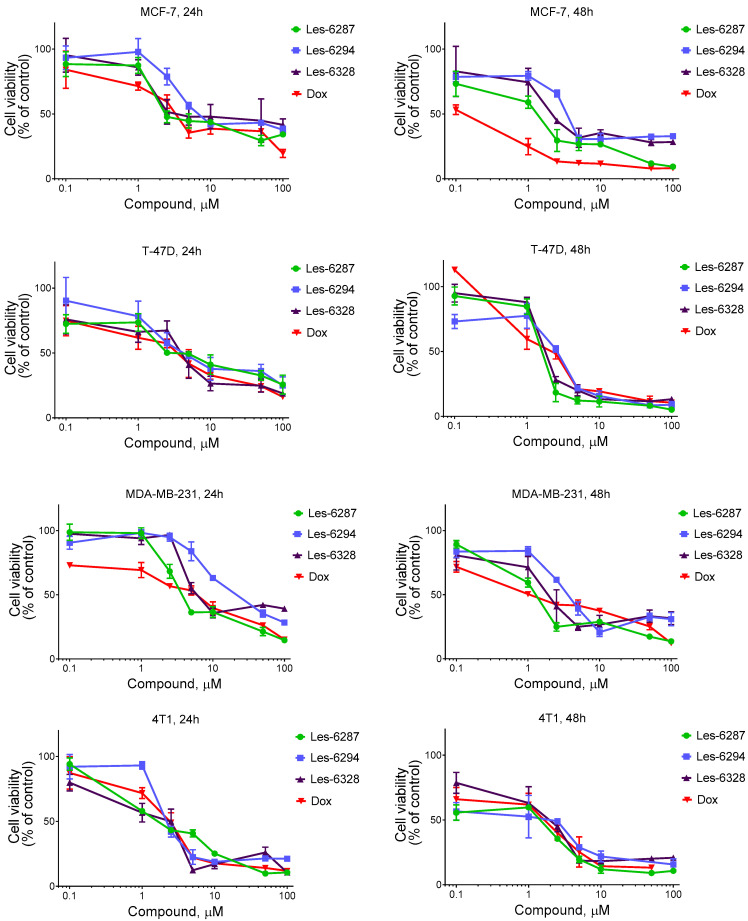

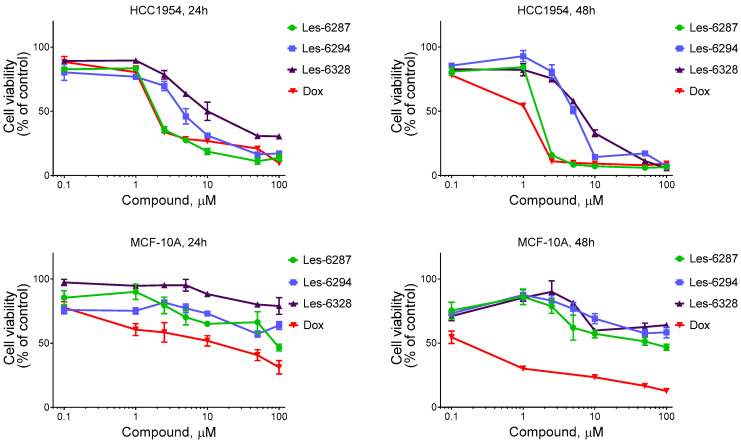
The derivatives **Les-6287**, **Les-6294**, **Les-6328**, and the reference drug (doxorubicin, Dox) affected the metabolic activity of breast carcinoma MCF-7, T-47D, MDA-MB-231, 4T1, and HCC1954 cells, and normal human breast epithelial MCF-10A cells after 24 and 48 h of their treatment. The data of the MTT assay are presented as M  ±  SD, n = 3.

**Les-6287**, **Les-6294**, and **Les-6328** compounds demonstrated high metabolic inhibitory activity towards breast carcinoma MCF-7, T-47D, MDA-MB-231, 4T1, and HCC1954 cells of different types (estrogen and progesterone receptor-positive, triple-negative, and HER2-positive, respectively; Figure 2, Table 2). After 24 h of exposure, the IC_50_ of **Les-6287** was 2.34 ± 0.16 µM in MCF-7 cells, 3.86 ± 0.24 µM in MDA-MB-231 cells, and 2.52 ± 0.65 µM in HCC1954 cells. After 48 h of exposure, the IC_50_ of **Les-6287** was 1.43 ± 0.18 μM for MCF-7 cells, 1.37 ± 0.15 μM for MDA-MB-231 cells, and 2.25 ± 0.64 μM in HCC1954 cells. The IC_50_ of compound **Les-6294** was 6.74 ± 0.64 μM after 24 h of MCF-7 cell treatment and 3.54 ± 0.14 μM after 48 h of cell treatment. Following 24 h of exposure, the IC_50_ of compound **Les-6294** was 21.85 ± 9.92 μM for MDA-MB-231 cells and 3.72 ± 0.22 μM following 48 h of cell treatment. The **Les-6294** compound demonstrated IC_50_ values of 4.53 ± 0.18 μM and 5.01 ± 0.23 μM after 24 and 48 h of HCC1954 cell treatment, respectively. After 24 h of exposure, the IC_50_ was 3.26 ± 0.40 μM under the **Les-6328** toward MCF-7 cells, 6.09 ± 0.33 μM toward MDA-MB-231 cells, and 9.91 ± 0.17 μM toward HCC1954 cells. After 48 h of exposure, the IC_50_ of 2.18 ± 0.19 μM was found under the **Les-6328** treatment of MCF-7 cells, 2.01 ± 0.12 μM under its treatment of MDA-MB-231 cells, and 6.40 ± 0.25 μM under its treatment of HCC1954 cells. The IC_50_ of the **Les-6287** compound was 3.11 ± 0.19 μM after 24 h of the T-47D cell treatment and 1.74 ± 0.25 μM after 48 h of cell treatment. Following 24 h of exposure, the IC_50_ of the **Les-6294** compound was 4.48 ± 0.51 μM for these cells and 2.66 ± 0.21 μM following 48 h of cell treatment. The IC_50_ of compound **Les-6328** was 4.08 ± 0.56 μM after 24 h of the T-47D cell treatment and 1.97 ± 0.58 μM after 48 h of cell treatment. After 24 h of exposure, the IC_50_ of **Les-6287** was 1.60 ± 0.14 µM in 4T1 cells, the IC_50_ of **Les-6294** was 2.20 ± 0.19 µM, and the IC_50_ of **Les-6328** was 2.25 ± 0.34 μM. After 48 h of exposure, the IC_50_ of **Les-6287** was 1.62 ± 0.21 μM for 4T1 cells, 2.22 ± 0.25 μM for **Les-6294**, and 1.94 ± 0.15 µM for **Les-6328**. It should be noted that the IC_50_ values for the studied hybrid molecules and doxorubicin were similar for the MCF-7, MDA-MB-231, and HCC1954 cells.

Thus, MDA-MB-231 and 4T1 triple-negative breast carcinoma cells, MCF-7 and T-47D estrogen and progesterone receptors positive, HER2 negative, and HCC1954 HER2 positive breast carcinoma cells were sensitive to the action of **Les-6287**, **Les-6294**, and **Les-6328** with IC_50_ levels of 1.37–21.85 µM at their treatment for 24 and 48 h. The hydroxy-substituted hybrid **Les-6287** was more active in the studied breast carcinoma cells. At the same time, related halogen-substituted molecules **Les-6328** and **Les-6294** had a lower inhibitory effect on studied breast carcinoma cells compared with **Les-6287**.

In the case of normal breast cells of the MCF-10A line, **Les-6287**, **Les-6294**, and **Les-6328** possessed lower cytotoxicity than in the case of breast carcinoma cells. The IC_50_ values for **Les-6287** were 93.01 ± 2.29 µM and 64.58 ± 0.68 µM after 24 and 48 h of exposure, respectively. The compounds **Les-6294** and **Les-6328** did not reach their IC_50_ at 100 μM (Figure 2, Table 2). In turn, the IC_50_ of Dox was 15.91 ± 0.91 µM after 24 h of MCF-10A cell treatment and 0.23 ± 0.05 μM after 48 h of cell treatment.

### 3.2. Studied Derivatives Inhibit the Formation of Colonies of Breast Cancer Cells

The biological efficiency of the studied compounds was determined using the clonogenic assay. An important factor in gauging an agent’s anticancer efficacy is its capacity to create colonies of new cells. This procedure is used to estimate the percentage of tumor cells that can still form colonies, a crucial phenotypic trait of tumor cells [26].

**Figure 3:**
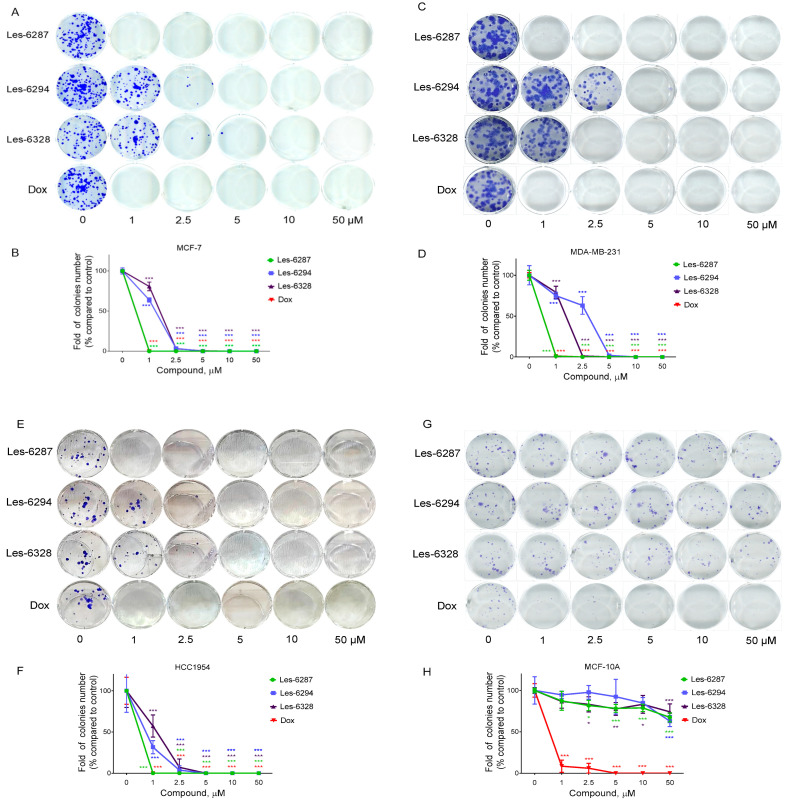
The effect of **Les-6287**, **Les-6294**, **Les-6328**, and doxorubicin (Dox) on the clonogenic ability of breast carcinoma MCF-7, MDA-MB-231, and HCC1954 cells and normal human breast epithelial MCF-10A cells under 14 days of cell exposure: The representative pictures of the formed colonies (**A**,**C**,**E**,**G**); and the numbers of the formed colonies of treated cells (**B**,**D**,**F**,**H**). Data are presented as the mean ± SD, n = 4. * *p* < 0.05; ** *p* < 0.01; *** *p* < 0.001 compared to control (non-treated) cells.

The **Les-6287** at 1 µM totally inhibited the formation of colonies of MCF-7, MDA-MB-231, and HCC1954 cells (Figure 3, Table 3). Doxorubicin demonstrated a similar effect on the formation of colonies of MCF-7, MDA-MB-231, and HCC1954 cells as **Les-6287** did. **Les-6294** and **Les-6328** demonstrated a less prominent ability to inhibit the formation of colonies of these cells. **Les-6294** and **Les-6328** at 5 µM totally inhibited the formation of colonies of MCF-7 cells. We did not observe the growth of MDA-MB-231 colonies after their exposure to **Les-6294** at 5 µM and **Les-6328** at 2.5 µM. The IC_50_ of **Les-6294** was 0.67 ± 0.08 µM, and the IC_50_ of **Les-6328** was 1.19 ± 0.03 µM for these cells. **Les-6294** and **Les-6328,** at a concentration of 2.5 µM, practically completely inhibited the formation of colonies of HCC1954 cells. The IC_50_ of **Les-6294** was 3.05 ± 0.03 µM, and the IC_50_ of **Les-6328** was 1.54 ± 0.02 µM for this cell line (Figure 3, Table 3).

Three studied derivatives slightly inhibited colony formation in normal human breast epithelial MCF-10A cells without reaching the IC_50_ at 50 µM (Figure 3, Table 3). **Les-6328** at a concentration of 50 µM inhibited the formation of colonies in these cells by 25.87%, **Les-6287** by 32.57%, and **Les-6294** by 37.12%.

### 3.3. The Effect of Studied Derivatives on the DNA Biosynthesis Process in Breast Carcinoma and Normal Breast Epithelial Cells

It should be noted that compound **Les-6287** was the most active among the studied derivatives. It was used further for in-depth evaluation of the anticancer activity towards human breast carcinoma MCF-7, MDA-MB-231, and HCC1954 cells.

**Figure 4:**
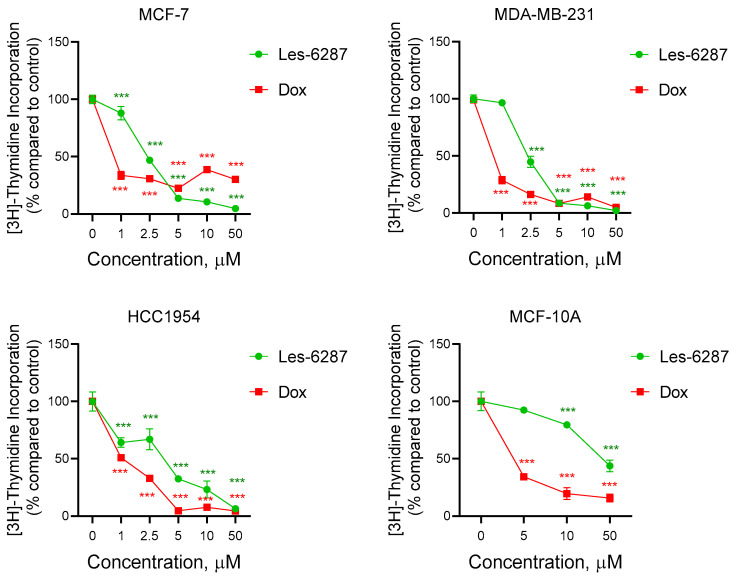
The incorporation of [^3^H]-thymidine into the DNA of MCF-7, MDA-MB-231, HCC1954, and MCF-10A cells under the 24 h effect of **Les-6287** and doxorubicin. The data are presented as M ± SD, n = 3. *** *p* < 0.001 compared to control (non-treated) cells.

The impact of the most active compound in MTT and clonogenic assays was therefore examined on DNA biosynthesis to identify the mechanism underlying their growth inhibition effect on human breast carcinoma cells of the MCF-7, MDA-MB-231, and HCC1954 lines and normal human breast epithelial cells of the MCF-10A line. The tested derivative inhibited DNA synthesis in breast cancer cell lines in a dose-dependent manner (Figure 4, Table 4). In MCF-7 and MDA-MB-231 cells, **Les-6287** exhibited similar DNA synthesis inhibitory effects, with IC_50_ values of 2.37 ± 0.02 µM and 2.32 ± 0.04 µM, respectively. This compound had a lower effect on DNA synthesis in HCC1954 cells, with an IC_50_ value of 3.67 ± 0.09 µM. For MCF-10A cells, **Les-6287** possessed a less pronounced impact on DNA biosynthesis, and the IC_50_ value for this cell line was higher than that observed for breast cancer cells. The IC_50_ value of **Les-6287** was 43.54 ± 1.16 µM for MCF-10A cells. The doxorubicin exhibited a higher effect on DNA biosynthesis in breast carcinoma and normal breast epithelial cells (Figure 4, Table 4).

### 3.4. **Les-6287** Induces Apoptosis by Extrinsic and Intrinsic Pathways

**Figure 5:**
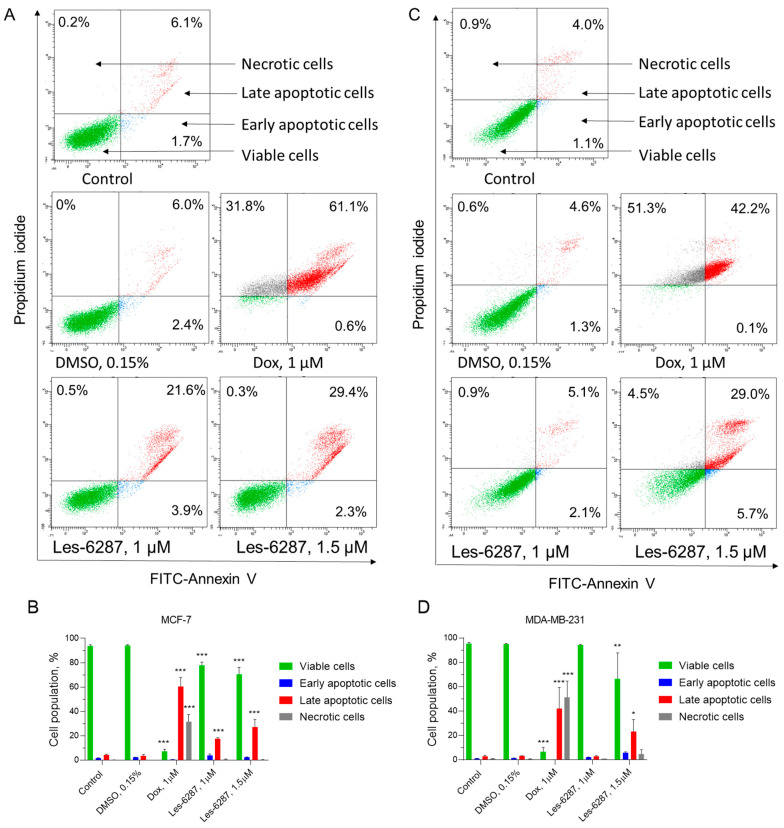
Flow cytometry analysis of human breast carcinoma MCF-7 (**A**,**B**) and MDA-MB-231 (**C**,**D**) cells after 24 h of incubation with **Les-6287** (1.0 μM and 1.5 μM), doxorubicin (1.0 μM), and the DMSO (0.15% corresponding to the solvent concentration at 1.5 μM of compound **Les-6287**) and subsequent staining with Annexin V and Propidium Iodide. Data are presented as the mean ± SD, n = 4. * *p* < 0.05; ** *p* < 0.01; *** *p* < 0.001 compared to the control (non-treated) cells.

**Les-6287** in 1 and 1.5 µM doses induced apoptosis in MCF-7 and MDA-MB-231 cell lines (Figure 5). In MCF-7 cells, 3.85 ± 1.20% of early apoptotic, 21.60 ± 0.78% of late apoptotic, and 0.50 ± 0.24% of necrotic cells were observed under **Les-6287** at 1 µM treatment, 2.30 ± 0.42% of early apoptotic, 29.35 ± 6.43% of late apoptotic, and 0.30 ± 0.11% of necrotic cells under **Les-6287** at 1.5 µM treatment. It should be noted that **Les-6287** did not affect the necrotic cell level in MCF-7 cells. We detected 2.10 ± 0.14% of early apoptotic, 5.10 ± 0.49% of late apoptotic, and 0.90 ± 0.02% of necrotic MDA-MB-231 cells after 24 h of incubation with a 1 µM concentration of **Les-6287**. In contrast, after cell incubation with a 1.5 µM concentration of **Les-6287**, 5.65 ± 0.92% of early apoptotic, 29.00 ± 9.76% of late, and 4.50 ± 1.82% of necrotic cells were detected. In the case of the control MDA-MB-231 cells, 1.10 ± 0.14% of early apoptotic, 3.95 ± 0.85% of late apoptotic, and 0.85 ± 0.21% of necrotic cells were detected. The doxorubicin-induced appearance of 0.55 ± 0.07% and 0.10 ± 0.01% of early apoptotic cells, 60.60 ± 7.35% and 42.20 ± 17.25% of late apoptotic cells, and 31.75 ± 5.59% and 51.25 ± 3.36% of necrotic cells in MCF-7 and MDA-MB-231 cells, respectively. As for the DMSO (0.15%), it did not cause changes in the number of early, late apoptotic, and necrotic cells, whose values were 2.35 ± 0.07%, 3.6 ± 0.99%, and 6.0 ± 1.2% for MCF-7 and 1.25 ± 0.21%, 3.25 ± 0.07%, and 0.60 ± 0.28% for MDA-MB-231 cells, respectively (Figure 5).

A reduction in mitochondrial membrane potential (ΔΨm) due to increased membrane permeability is associated with the early stages of apoptotic death that occur via the intrinsic pathway [30]. JC-1 fluorescent dye labeling was used to examine the impact of **Les-6287** on the intrinsic apoptotic pathway in breast carcinoma cells.

**Figure 6:**
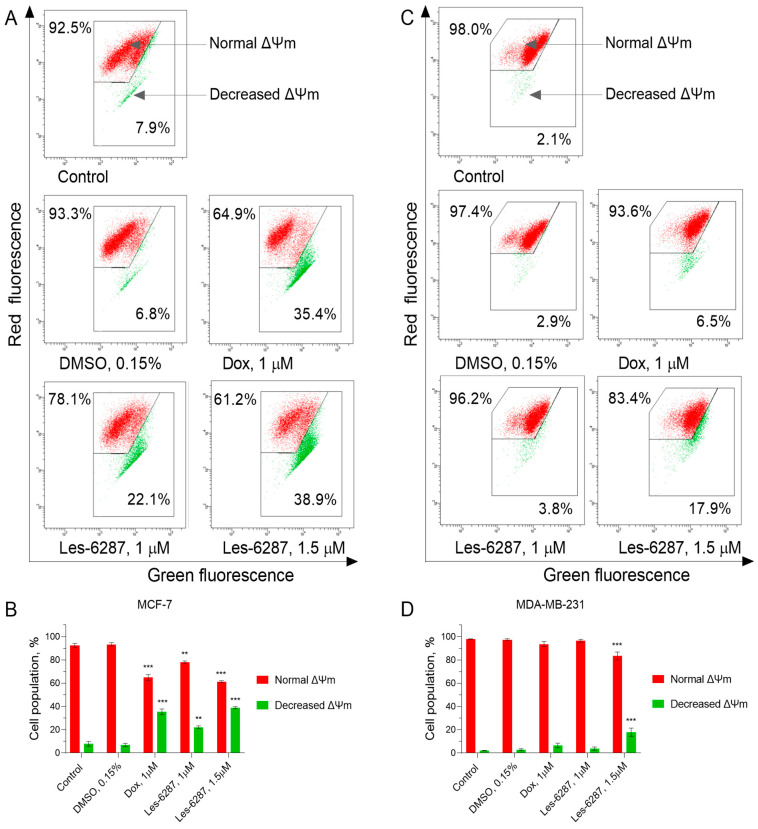
Flow cytometry analysis of the mitochondrial membrane potential changes (MMP, ΔΨm) in MCF-7 (**A**,**B**) and MDA-MB-231 (**C**,**D**) breast cancer cells after 24 h of incubation with **Les-6287** (1.0 μM and 1.5 μM), doxorubicin (1.0 μM), and the DMSO (0.15% corresponding the solvent concentration at 1.5 μM of compound **Les-6287**). Data are presented as the mean ± SD, n = 4. ** *p* < 0.01; *** *p* < 0.001 compared to the control (non-treated) cells.

As shown in Figure 6, **Les-6287** caused an increase in the percentage of cells with decreased mitochondrial membrane potential in MCF-7 and MDA-MB-231 cells. However, it was greater for MCF-7 cells: 21.00 ± 1.20% (**Les-6287**, 1.0 μM) and 38.85 ± 3.46% (**Les-6287**, 1.5 μM). For MDA-MB-231 cells, the values were as follows: 3.75 ± 1.34% **Les-6287** (1.0 μM) and 17.85 ± 3.46% **Les-6287** (1.5 μM).

**Figure 7:**
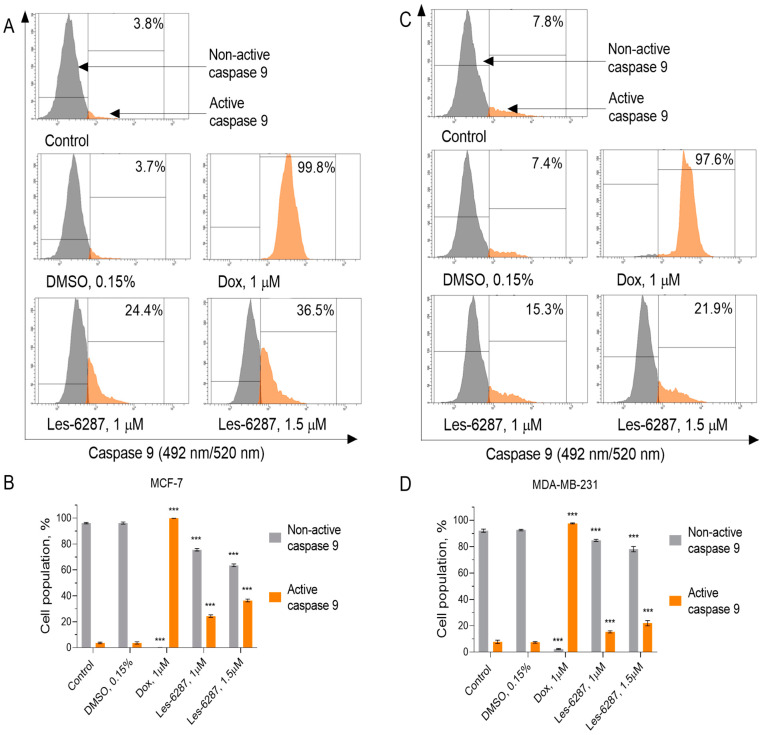
Flow cytometry analysis of the caspase-9 activity in the MCF-7 (**A**,**B**) and MDA-MB-231 (**C**,**D**) breast cancer cells after 24 h of incubation with **Les-6287** (1.0 μM and 1.5 μM), doxorubicin (1.0 μM), and DMSO (0.15% corresponding to the solvent concentration at 1.5 μM of compound **Les-6287**). Data are presented as the mean ± SD, n = 4. *** *p* < 0.001 compared to the control (non-treated) cells.

An appearance of pores and a related increase in mitochondrial membrane permeability result in the release of cytochrome *c* into the cytosol and apoptosome formation that triggers the activation of caspase 9 [28]. The assumption of the present assay was to evaluate caspase-9 activity in MCF-7 and MDA-MB-231 cancer cells after 24 h of incubation with the tested compounds. **Les-6287** increased caspase-9 activity in both line cells (Figure 7). The level of active caspase 9 in the MCF-7 cells increased to 24.40 ± 0.98%. (**Les-6287**, 1.0 μM) and 36.45 ± 1.05% (**Les-6287**, 1.5 μM) compared to the control group. In contrast, an increase in caspase-9 activity to 15.30 ± 0.80% (**Les-6287**, 1.0 μM) and 21.88 ± 2.00% (**Les-6287**, 1.5 μM) was observed in the MDA-MB-231 cell line. The data from the present experiment correlate well with changes in ΔΨm, confirming that apoptosis is induced via the intrinsic pathway.

**Figure 8:**
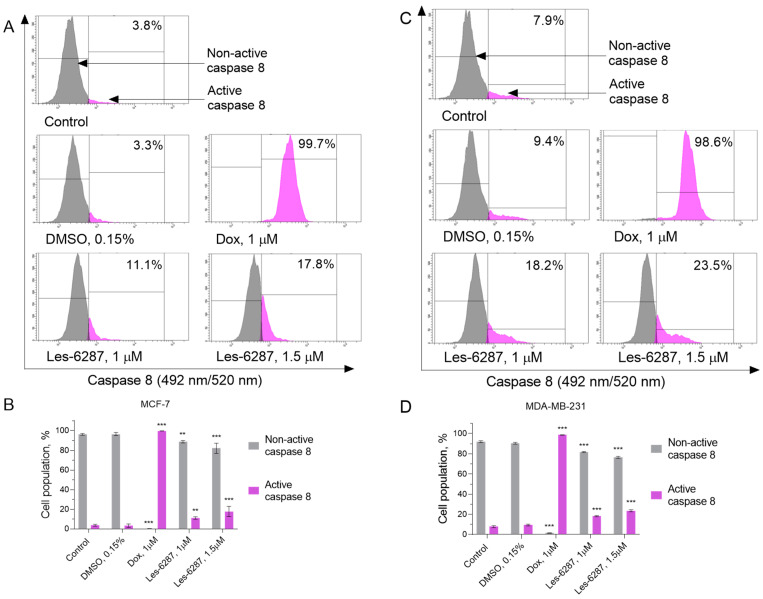
Flow cytometry analysis of the caspase-8 activity in the MCF-7 (**A**,**B**) and MDA-MB-231 (**C**,**D**) breast cancer cells after 24 h of incubation with **Les-6287** (1.0 μM and 1.5 μM), doxorubicin (1.0 μM), and DMSO (0.15% corresponding to the solvent concentration at 1.5 μM of compound **Les-6287**). Data are presented as the mean ± SD, n = 4. ** *p* < 0.01; *** *p* < 0.001 compared to the control (non-treated) cells.

**Figure 9:**
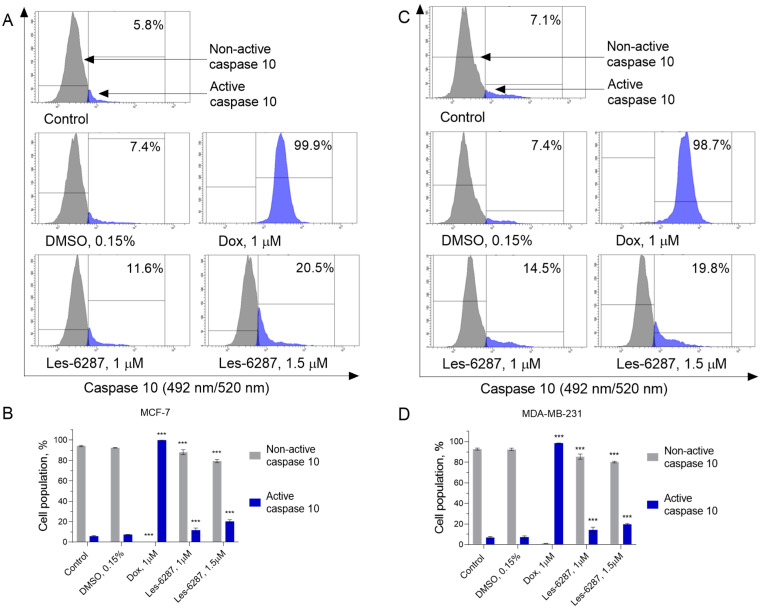
Flow cytometry analysis of the caspase-10 activity in the MCF-7 (**A**,**B**) and MDA-MB-231 (**C**,**D**) breast cancer cells after 24 h of incubation with **Les-6287** (1.0 μM and 1.5 μM), doxorubicin (1.0 μM), and DMSO (0.15% corresponding to the solvent concentration at 1.5 μM of compound **Les-6287**). Data are presented as the mean ± SD, n = 4. *** *p* < 0.001 compared to the control (non-treated) cells.

Activation of caspases 8 and 10 is a key process required to induce apoptosis via the extrinsic pathway [31]. Thus, we measured the caspase 8 and 10 activity in the MCF-7 and MDA-MB-231 cells after 24 h of treatment with the tested compounds (Figure 8 and Figure 9). After treatment with **Les-6287**, activation of the caspase 8 and 10 was observed in 11.08 ± 1.30% and 11.58 ± 2.30% (caspase 8 and 10, **Les-6287**, 1.0 μM) and 17.78 ± 5.19% and 20.50 ± 1.49% (caspase 8 and 10, **Les-6287**, 1.5 μM) of the MCF-7 cell population, respectively, while for MDA-MB-231, an increase in the active form of the caspase 8 and 10 was up to 18.23 ± 0.62% and 14.48 ± 2.53% (**Les-6287**, 1.0 μM) and 23.50 ± 1.03% and 19.78 ± 0.80% (**Les-6287**, 1.5 μM), respectively. These observations indicate that the results correspond well with those of the Annexin V/PI assay, demonstrating that the apoptosis process can be induced by an extrinsic pathway mediated by death receptors.

**Figure 10:**
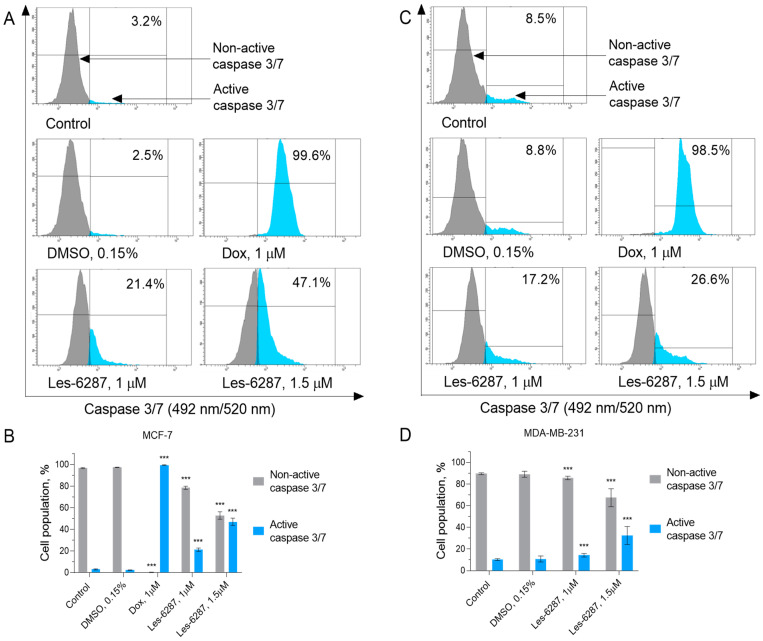
Flow cytometry analysis of the caspase 3/7 activity in the MCF-7 (**A**,**B**) and MDA-MB-231 (**C**,**D**) breast cancer cells after 24 h of incubation with **Les-6287** (1.0 μM and 1.5 μM) and DMSO (0.15% corresponding to the solvent concentration at 1.5 μM of compound **Les-6287**). Data are presented as the mean ± SD, n = 4. *** *p* < 0.001 compared to the control (non-treated) cells.

During apoptosis, the fusion of the extrinsic and intrinsic pathways into one common pathway takes place. At this point, the cascade of executioner caspases (caspases 3, 6, 7) begins, and the transition into the final phase of programmed cell death [32]. We assessed the activity of caspase 3/7 in MCF-7 and MDA-MB-231 cells after treatment with tested compounds (Figure 10). The percentage of the MCF-7 cells with active caspase 3/7 reached 21.38 ± 1.50% (**Les-6287**, 1.0 μM) and 47.08 ± 3.40% (**Les-6287**, 1.5 μM) of the tested cell population. For the MDA-MB-231 cancer cells, the value oscillated between 17.20 ± 1.58% (**Les-6287**, 1.0 μM) and 26.63 ± 8.32% (**Les-6287**, 1.5 μM). Doxorubicin activated caspases 8, 9, 10, and 3/7 in a more pronounced manner than the studied derivatives did (data are not presented). This investigation shows that all previous results were consistent with each other, indicating the ability of **Les-6287** to induce apoptosis, and this process follows two pathways.

### 3.5. Compounds Inhibit Autophagy in Breast Cancer Cells

Due to the correlation between the degree of autophagy and the posttranslational alterations of LC3A/B and the regulation of Beclin-1, we used PCR and ELISA to examine these proteins.

**Figure 11:**
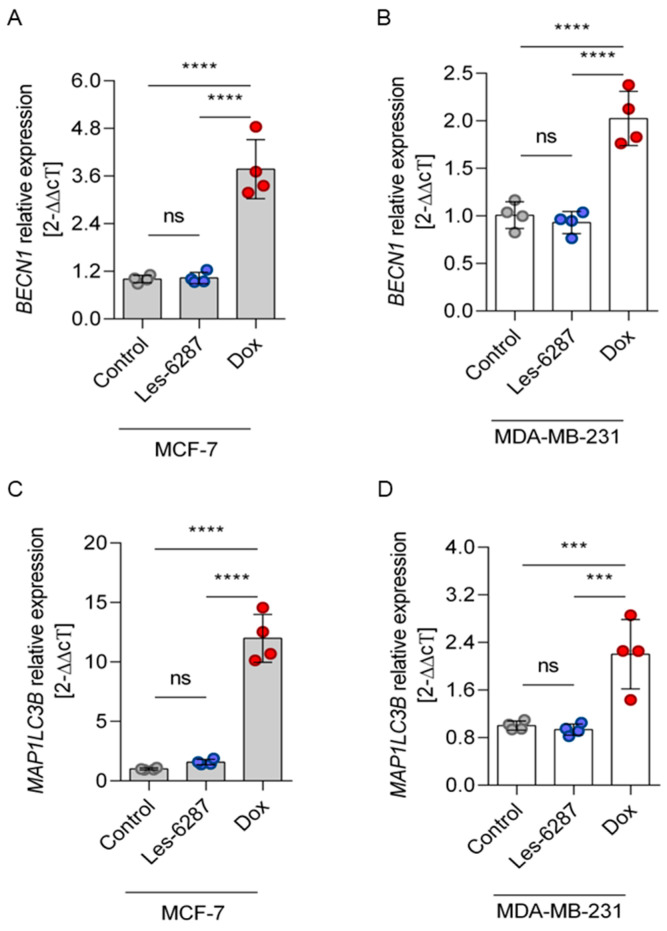
The expression of *BECN1* and *MAP1LC3B* genes in the MCF-7 and MDA-MB-231 breast cancer cells after 24 h of incubation with **Les-6287** and the doxorubicin at 1 µM concentration: *BECN1* expression in MCF-7 (**A**) and MDA-MB-231 (**B**) cells; *MAP1LC3B* expression in MCF-7 (**C**) and MDA-MB-231 (**D**) cells. Data are presented as M ± SD from three independent experiments performed in duplicate. *** *p* < 0.001; **** *p* < 0.0001; ns—non-significant changes.

As demonstrated in Figure 11, in MCF-7 and MDA-MB-1 cells treated for 24 h with doxorubicin (1 µM), the expression of both *MAP1LC3b* and *BECN1* genes was significantly increased compared to control cells. In contrast, incubation of MCF-7 and MDA-MB-1 with **Les-6287** did not affect *MAP1LC3B* and *BECN1* expression.

**Figure 12:**
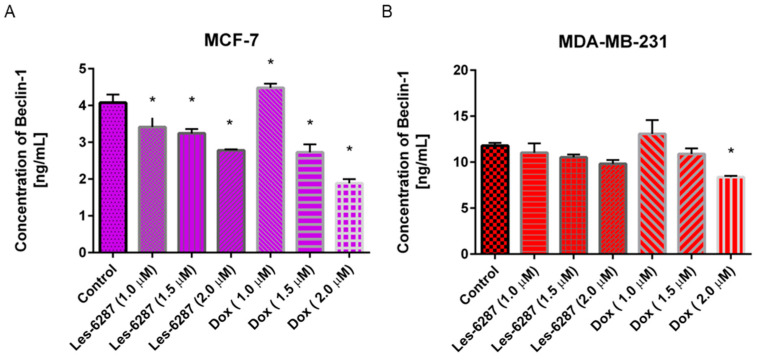
The concentration of Beclin-1 in the MCF-7 (**A**) and the MDA-MB-231 (**B**) cells after 24 h of incubation with **Les-6287** and doxorubicin at 1 µM, 1.5 µM, and 2 µM concentrations. Data are presented as M ± SD from three independent experiments performed in duplicate. * *p* < 0.05 compared to control (non-treated) cells.

Figure 12 demonstrates a dose-dependent inhibitory effect of **Les-6287** and the reference drug, doxorubicin, on the concentration of Beclin-1 in MCF-7 and MDA-MB-231 cells. In the untreated control MCF-7 and MDA-MB-231 cells, the concentration of Beclin-1 was 4.08 ng/mL and 11.79 ng/mL, respectively. The most significant decrease in Beclin-1 concentration was observed in doxorubicin—1.89 ng/mL at 2 μM in MCF-7 cells. Additionally, 24 h incubation with **Les-6287** resulted in a reduction in Beclin-1 concentration to 2.78 ng/mL at 2 μM in the MCF-7 cells and 9.83 ng/mL in the MDA-MB-231 cells.

**Figure 13:**
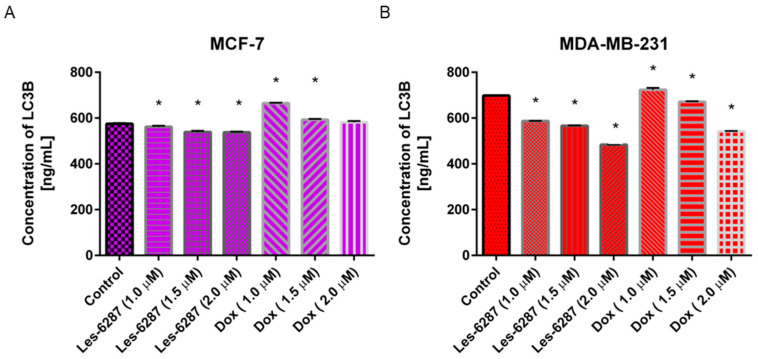
The concentration of LC3B in the MCF-7 (**A**) and MDA-MB-231 (**B**) cells after 24 h of incubation with **Les-6287** and the reference drug (doxorubicin) at 1 µM, 1.5 µM, and 2 µM concentrations. Data are presented as M ± SD from three independent experiments performed in duplicate. * *p* < 0.05 compared to control (non-treated) cells.

As demonstrated in Figure 13, the inhibitory activity towards microtubule-associated protein 1A/1B light chain 3B (LC3B) was observed for all three doses of **Les-6287** (1.0, 1.5, and 2.0 μM) in both analyzed breast cancer cell lines. For **Les-6287**, the concentration of this biomarker of autophagy was reduced from 574.84 ng/mL in the control MCF-7 cells to 537.74 ng/mL. In the MDA-MB-231 breast cancer cells, the concentration of LC3B in the non-treated control was 698.25 ng/mL. **Les-6287** at concentrations of 2 μM reduced the level of LC3B to 483.50 ng/mL.

Thus, autophagy is reduced in breast carcinoma MCF-7 and MDA-MB-231 cells under the action of **Les-6287**.

### 3.6. **Les-6287** Decreases the Concentration of MMP-2, MMP-9, and ICAM-1 Proteins, Which Are Involved in Metastasis and Invasion

MMP-2 is considered a prognostic marker in many carcinomas. Talvensaari-Mattila et al. proved that MMP-2 is associated with survival in breast carcinoma and correlates to shortened survival [33].

**Figure 14:**
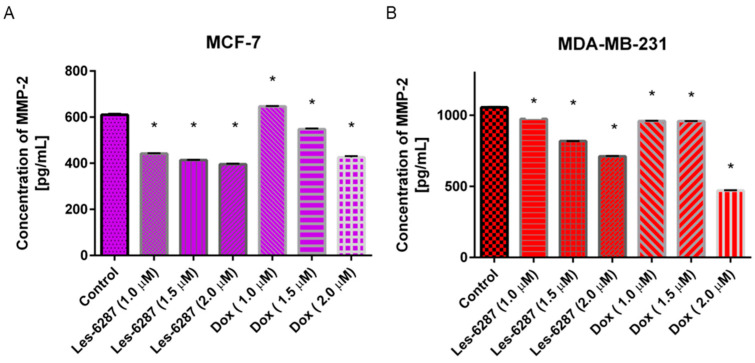
The concentration of MMP-2 in the MCF-7 (**A**) and MDA-MB-231 (**B**) cells after 24 h of incubation with **Les-6287** and doxorubicin at 1 µM, 1.5 µM, and 2 µM concentrations. Data are presented as M ± SD from three independent experiments performed in duplicate. * *p* < 0.05 compared with the control (non-treated) cells.

An inhibitory effect on MMP-2 was observed in all the tested samples in both examined breast cancer cell lines. As demonstrated in Figure 14, the most significant decrease was observed for Les-6287, from 610.57 pg/mL in untreated MCF-7 control cells to 395.91 pg/mL. The doxorubicin at 2 μM decreased the MMP-2 concentration to 425.40 pg/mL in MCF-7 cells. After incubation with **Les-6287** at 2 μM, the concentration of MMP-2 was lowered to 711.74 pg/mL in the MDA-MB-231 breast cancer cells, in comparison with the control, where the MMP-2 concentration was 1055.21 pg/mL. Doxorubicin (2 μM) caused a decline in the MMP-2 concentration to 470.57 pg/mL, respectively.

**Figure 15:**
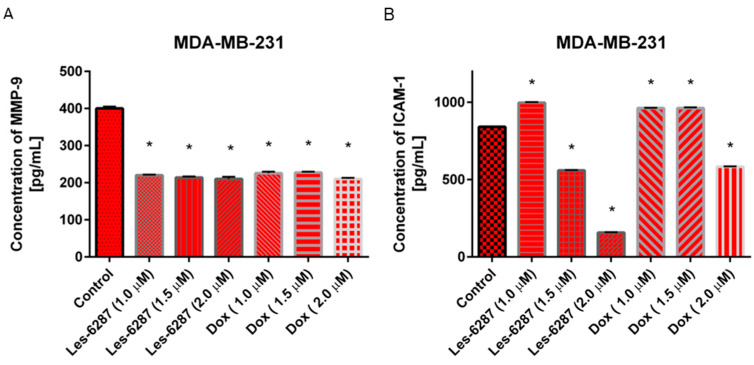
The concentration of MMP-9 (**A**) and ICAM-1 (**B**) in the MDA-MB-231 human breast cancer cells after 24 h of incubation with **Les-6287** and doxorubicin at 1 µM, 2 µM, and 2 µM concentrations. Data are presented as M ± SD from three independent experiments performed in duplicate. * *p* < 0.05 compared to the control (non-treated) cells.

Additionally, the concentrations of MMP-9 and ICAM-1 were measured in the MDA-MB-231 breast cancer cells after 24 h of incubation with **Les-6287,** as well as doxorubicin (Figure 15). We found that the MMP-9 and ICAM-1 concentrations in MCF-7 breast cancer cells were below the detection range. In the case of MMP-9, we proved that all examined doses of **Les-6287** and doxorubicin decreased MMP-9 concentrations. The most significant decrease in the ICAM-1 concentration (157.25 pg/mL) was observed after 24 h of incubation with **Les-6287** (2 µM). The doxorubicin at concentrations of 1.0 and 1.5 µM increased the ICAM-1 concentration, but the highest dose of this reference drug decreased the ICAM-1 concentration (583.04 pg/mL) compared to the control (841.55 pg/mL).

Thus, one can assume that **Les-6287** may inhibit the metastasis and invasion of breast carcinoma cells. It should be noted that these processes are key factors in cancer progression and are often associated with a poor prognosis [34,35].

## 4. Discussion

Heterocyclic compounds represent the chemical basis of >85% of the FDA-approved medicines [36]. Approximately 50% of those heterocycles have a hybrid structure composed of two-parent molecules (pharmacophores) that may affect different pharmacological targets [37]. Such a synergistic action of a hybrid molecule leads to enhanced pharmacological potential. In this study, we used the pyrrolidinedione–thiazolidinone hybrids, namely **Les-6287**, **Les-6294**, and **Les-6328**. Both components of these hybrids demonstrated medicinal effects and are used in various drugs and drug-like substances [38]. **Les-6287**, **Les-6294**, and **Les-6328** compounds were evaluated for their impact on the metabolic activity and survivability of breast carcinoma cells of MCF-7 and T-47D (ER+, PR+, HER2-), MDA-MB-231 and 4T1 (ER-, PR-, HER2-), and HCC1954 (ER-, PR-, HER2+) lines, and normal breast cells of the MCF-10A line.

The TNBC and receptor-positive breast cancer cell lines under study (MDA-MB-231, 4T1, MCF-7, T-47D, and HCC1954) were found to be sensitive to the toxic action of hybrid heterocyclic molecules (**Les-6287**, **Les-6294**, and **Les-6328**), with the half-maximal inhibitory concentration (IC_50_) ranging from 1.37 to 21.85 µM depending on the incubation time (24 or 48 h). We have shown that the hydroxy-substituted hybrid compound **Les-6287** exhibited the most pronounced cytotoxic activity (24 h) in MCF-7, T-47D, HCC1954, MDA-MB-231, and 4T1 cells with IC_50_ values of 2.34, 3.11, 2.52, 3.86, and 1.60 µM, respectively, compared to halogen-substituted derivatives **Les-6294** with IC_50_ values of 6.74, 4.48, 4.53, 21.85, and 2.20 μM, and **Les-6328** with IC_50_ values of 3.26, 4.08, 9.91, 6.09, and 2.25 μM, respectively. Both halogen-substituted derivatives, **Les-6294** (with chloro-substituent) and **Les-6328** (with bromo-substituent), showed almost the same activity. In our opinion, the presence of a hydroxy group in position 4 of the phenyl ring leads to a higher activity level. Thus, breast carcinoma cells of MCF-7, T-47D (ER+, PR+, HER2-), MDA-MB-231, 4T1 (ER-, PR-, HER2-), and HCC1954 (ER-, PR-, HER2+) lines were likewise sensitive to the action of **Les-6287** and **Les-6328** compounds. **Les-6294** demonstrated varying toxicity towards studied breast carcinoma cells at 24 h of treatment and comparable toxicity at the 48 h mark. It should be stressed that the cytotoxic activity of **Les-6287**, **Les-6294**, and **Les-6328** compounds was significantly higher for studied breast carcinoma cells than for normal human breast epithelial MCF-10A cells (IC_50_ > 64.58 μM).

The studied derivatives demonstrated selectivity toward the analyzed breast cancer cells, and the selectivity index (SI = IC_50_ for MCF-10A/IC_50_ for human breast tumor cells) for **Les-6287** was 24.10–47.13, **Les-6294**—4.57–37.59, and for **Les-6328**—10.09–50.76. More detailed dose–response studies are being planned to determine the therapeutic windows of the compounds.

In earlier research, Finiuk et al. (2022) discovered that the pyrrolidinedione–thiazolidinone hybrids **Les-6287** and **Les-6294** inhibited the metabolic activity of the MDA-231, MCF-7, cervical (HeLa), colon (HCT-116), and glioma (U251) cells, as well as of human leukemia (Jurkat) cells. The IC_50_ value of these derivatives ranged from roughly 1.3 to 9.2 µM [23]. The IC_50_ values of these derivatives were higher than 73.6 µM for normal human keratinocytes (HaCaT) and 29–67 µM for mitogen-activated lymphocytes collected from the peripheral blood of a healthy donor [24].

One important metric for estimating the anticancer activity of investigated drugs is the colony-forming capacity of tumor cells. The ability of tumor cells to form colonies may also be associated with their potential to metastasize or spread to other parts of the body [26]. The **Les-6287** compound at 1 µM effectively inhibited the formation of colonies of the MCF-7, MDA-MB-231, and HCC1954 breast cancer cells at a similar level as the well-known anticancer drug doxorubicin did. In this case, the **Les-6294** and **Les-6328** derivatives at a 1 µM dose had a weaker effect on the colony-forming ability of breast cells versus the **Les-6287**. Although at a 2.5–5 µM dose, the **Les-6294** and **Les-6328** heterocycles fully inhibited the formation of breast cancer colonies. It is important to note that the studied hybrid heterocyclic molecules did not show cytotoxic activity towards normal MCF-10A breast epithelial cells, and the IC_50_ value of the most effective **Les-6287** compound was only 93.01 ± 2.29 µM under 24 h of treatment. In addition, the studied derivatives very weakly inhibited the ability of normal human breast epithelial cells to form colonies, and a significant number of MCF-10A colonies were observed under treatment with **Les-6287**, **Les-6294**, and **Les-6328** (50 μM).

DNA biosynthesis is a vital process involved in the proliferation of all mammalian cells. An enhanced metabolism of cancer cells causes them to progress more quickly and uncontrollably, leading to the rapid proliferation of diseased tissues. Because of this, substances with anticancer potential should exhibit antiproliferative properties [39]. The **Les-6287** compound inhibited DNA synthesis in the MCF-7, MDA-MB-231, and HCC1954 cell lines, with IC_50_ in the range of 2.32–3.67 µM. A considerably decreased effect of **Les-6287** on the suppression of DNA synthesis was observed in the treated MCF-10A cells (IC_50_ equaled 43.54 µM).

Two cell lines, namely the hormone-receptor-positive MCF-7 cells, which were more susceptible to the chemicals, and triple-negative MDA-MB-231 breast cancer cells, which were more resistant, were used to examine the mechanisms of action of the **Les-6287**.

It is known that TNBC is refractory to targeted hormone receptor-dependent therapies and demonstrates a more aggressive clinical course [14]. Thus, the revealed cytotoxicity of the **Les-6287** compound against MDA-MB-231 cells belonging to the TNBC type displaces the potential of this agent in targeted chemotherapy. In addition, the role of the immune system realized via programmed death ligand 1 (PD-L1) should be taken into consideration. PD-L1 participates in an inhibitory immune checkpoint axis in TNBC [40]. Its expression in the TNBC allows one to propose the application of PD-L1 inhibitors in combination with novel heterocyclic compounds to enhance anticancer chemotherapy through simultaneous targeting of cancer cell viability and the immune microenvironment.

Apoptosis plays a crucial role in the cell death of cancerous cells. A series of caspase activation events during apoptosis facilitate signal transduction and cellular death [41]. A reduction in mitochondrial membrane potential, the release of cytochrome c, and an increase in caspase-9 and caspase-3 activity are all seen in the intrinsic pathway of apoptosis. The early indicator of apoptosis is thought to be the loss of mitochondrial transmembrane potential (ΔΨm). Conversely, executive caspase 3 and caspase 7 are triggered by a signal from activated caspases 8, 9, and 10 in both the intrinsic and extrinsic pathways of apoptosis [42].

**Figure 16:**
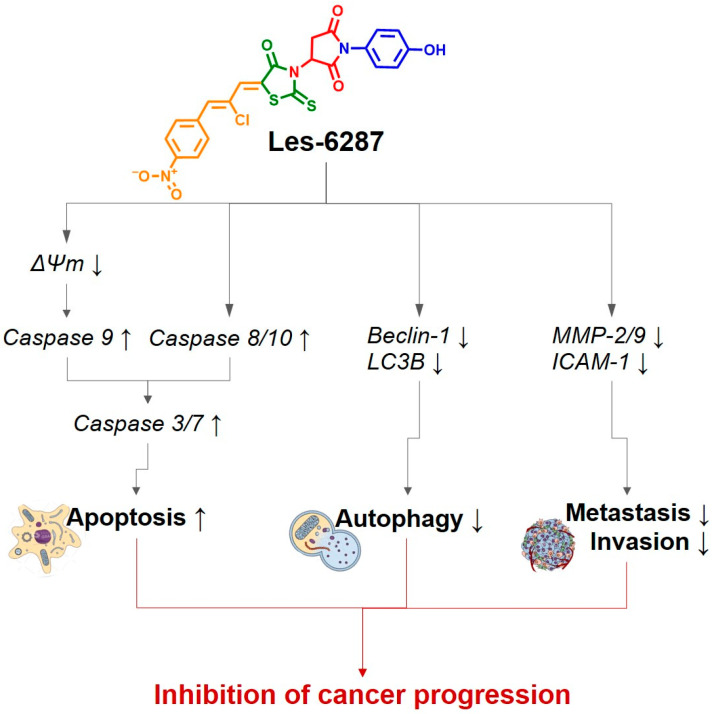
General schema of **Les-6287** action on breast tumor cells.

Our analysis of caspase-9 activity revealed a marked rise in the activity of this protein under the **Les-6287** treatment of the MCF-7 and MDA-MB-231 cells. Caspase-3/7 activity increased in correlation with its rise. Notably, the MCF-7 breast carcinoma cells lack caspase 3 [43], indicating that the activation of caspase 7 is linked to the functioning of the executioner caspases. Thus, **Les-6287** induced the intrinsic route of apoptosis. The activation of caspases 8 and 10 takes place in the extrinsic pathway [41]. **Les-6287** activated caspases 8 and 10 in MCF-7 and MDA-MB-231 cells. This might indicate that it also caused the extrinsic pathway of programmed cell death. These findings can also be linked to our outcomes, which are that **Les-6287** influences the upregulation of active caspase 3/7 in studied cells. Thus, **Les-6287** induced the extrinsic and intrinsic apoptotic pathways in breast tumor cells (Figure 16).

We did not reveal significant values of necrosis in breast cancer cells treated with the **Les-6287** compound—0.30–0.50% of the necrotic MCF-7 cells and 0.90–4.50% of the necrotic MDA-MB-231 cells, while the doxorubicin-induced necrotic cells were 31.75 and 51.25% in the MCF-7 and MDA-MB-231 lines, respectively.

Autophagy is a process that can be dysregulated in many diseases, including cancer, which plays a dichotomous role by inhibiting or initiating tumor progression. Understanding the molecular mechanism of this self-degradation system is important to developing new targeted therapies. More evidence supports the idea that inhibiting autophagy is a promising strategy to fight advanced cancer [44]. We demonstrated that the tested compound decreased Beclin-1 and LC3B concentrations in MCF-7 and MDA-MB-231 breast cancer cells. Doxorubicin increased the expression of the *MAP1LC3B* and *BECN1* genes and elevated the concentrations of Beclin-1 and LC3B in the MCF-7 and MDA-MB-231 breast cancer cells.

It has been demonstrated that caspase-3 activation inhibits autophagy by cleaving Beclin-1 [45]. Thus, the cleavage of this protein under the action of **Les-6287** may have a role in autophagy suppression during apoptosis in breast carcinoma cells.

Similar results were obtained by Buzun et al. (2022), who reported that the Ciminalum–4-thiazolidinone hybrid induced the intrinsic and extrinsic apoptotic pathways (decreased ΔΨm, increase in caspase-9 and caspase-8 concentration) in the MCF-7 and MDA-MB-231 cells. This derivative caused a decrease in concentrations of Beclin-1 and LC3B and inhibited autophagy in both analyzed breast carcinoma cells [22]. Campos et al. (2022) demonstrated that 2-imino-4-thiazolidinones have proapoptotic activity due to a significant stimulation of caspases-3/7 in C6 glioblastoma cells [16].

In many studies, it has been confirmed that matrix metalloproteinases (MMPs) play pivotal roles in metastasis and are associated with survival in several cancers [46]. Matrix metalloproteinase-9 (MMP-9) represents a major member of the MMP family, and its overexpression was confirmed in many human malignant tumors such as gastric, liver, lung, bone, skin, and breast [47]. Jiang and co-authors reported that the overexpression of MMP-2 and MMP-9 in cancer cells was linked to poor survival, lymph node metastasis, and larger tumor size [46]. The concentrations of MMP-2 and MMP-9 in breast cancer cells declined under treatment with the **Les-6287,** as well as the doxorubicin.

Chen et al. demonstrated that ICAM-1 in TNBC is strongly involved in tumorigenesis and metastasis. Its high expression was found to correlate with a poor prognosis in TNBC. ICAM-1 promotes EMT via integrin-mediated TGF-β/EMT signaling in TNBC by regulating the expression of genes such as MMPs. The authors proved that silencing ICAM-1 expression significantly inhibited bone metastasis in tumor-bearing mice [48]. These findings support the hypothesis that ICAM-1 targeted therapy in triple-negative breast cancer patients is rational.

In our study, we confirmed the inhibitory effect of **Les-6287** on the ICAM-1 concentration in the MDA-MB-231 breast cancer cell line belonging to TNBC. The inhibitory effect was almost four times stronger than that of the reference compound—doxorubicin.

The results obtained at the ELISA measurements proved that the **Les-6287** compound can decrease the concentration of MMP-2, MMP-9, and ICAM-1 proteins involved in metastasis and poor prognosis in breast cancer patients (Figure 16).

Therefore, our preclinical study indicates the prospect of further research into the hybrid pyrrolidinedione–thiazolidinone molecules as promising cancer-targeted compounds for the treatment of immunohistochemically different subtypes of breast cancer. We plan long-term studies to reveal cell responses for a better understanding of the temporal effects of treatments with these heterocyclic agents. These hybrid molecules will be combined with standard and new chemotherapeutic agents that could enhance efficacy and reduce drug resistance. Further work should include a study of the therapeutic potential and safety profile in vivo of the most effective hybrid heterocyclic derivative, particularly **Les-6287**, in breast cancer-bearing animals, followed by a detailed study of the pharmacokinetics and pharmacodynamics of this compound in the organisms of treated animals. If animal studies are successful, the most effective hybrid heterocyclic molecule (i.e., **Les-6287**) might be used as a candidate for the synthesis of conjugates of antibodies with pyrrolidinedione–thiazolidinone agents. The carriers (i.e., biomimetic or nanoscale carriers) or integrating miRNA-based gene therapy could be combined with the antitumor effects of the **Les-6287** compound to improve the therapeutic index and reduce off-target effects. In the distant future, personalized medical techniques, such as single-cell sequencing, may be used to identify patients who would benefit the most from such combination therapies.

## 5. Conclusions

We have shown that novel hybrid pyrrolidinedione–thiazolidinone molecules **Les-6287, Les-6294,** and **Les-6328** possessed cytotoxicity towards human breast carcinoma cells of MCF-7, T-47D, MDA-MB-231, and HCC1954 lines and murine breast cancer 4T1 cells that differ in their genotype (depending on ER, PR, and HER2 expression). They demonstrated low toxicity towards normal human breast cells in MCF-10A. **Les-6287** completely prevented MCF-7 (ER+, PR+, HER2-), MDA-MB-231 (ER-, PR-, HER2-), and HCC1954 (ER-, PR-, HER2+) cells from forming colonies at 1 µM; the **Les-6294** and **Les-6328** compounds did the same at 2.5 or 5 µM doses. Conversely, at 50 µM, none of the investigated derivatives reached the IC_50_ and only mildly hindered the formation of colonies of normal human breast epithelial MCF-10A cells. **Les-6287** caused apoptosis in the MCF-7 and MDA-MB-231 cells. Both an intrinsic pathway—shown as a rise in caspase-9 activity and a decrease in the mitochondrial potential—and an extrinsic pathway with the activation of caspases 8 and 10 are followed in this process. Furthermore, elevated activity of common executioner caspases 3/7 for both apoptotic pathways was found. In addition, the derivative under investigation reduced the concentrations of Beclin-1 and LC3B and hindered autophagy. **Les-6287** reduced the concentration of MMP-2, MMP-9, and ICAM-1, all of which are linked to metastasis and a poor prognosis in breast cancer patients. Thus, the developed hybrid pyrrolidinedione–thiazolidinones are promising substances for targeting human breast carcinoma cells. The obtained outcomes serve as the basis for precise research into the mechanisms underlying the therapeutic activity of **Les-6287**, **Les-6294**, and **Les-6328** in vivo for breast cancer of different subtypes.

## Figures and Tables

**Table 1:** The list of PrimePCR SYBR Green assays used in the study.

Assay	Target Species	Catalog Number	Manufacturer
MAP1LC3B	Human	qHsaCEP0041298	Bio-Rad
BECN1	Human	qHsaCID0016032	Bio-Rad
GAPDH	Human	qHsaCEP0041396	Bio-Rad

**Table 2:** The IC_50_ values of studied compounds targeting cells in MTT assay.

Cell Line	Timepoint, h	IC_50_, µM (M ± SD)			
		Les-6287	Les-6294	Les-6328	Dox
MCF-7	24	2.34 ± 0.16	6.74 ± 0.64	3.26 ± 0.40	3.47 ± 0.49
	48	1.43 ± 0.18	3.54 ± 0.14	2.18 ± 0.19	0.18 ± 0.07
T-47D	24	3.11 ± 0.19	4.48 ± 0.51	4.08 ± 0.56	3.66 ± 0.33
	48	1.74 ± 0.25	2.66 ± 0.21	1.97 ± 0.58	2.31 ± 0.24
MDA-MB-231	24	3.86 ± 0.24	21.85 ± 9.92	6.09 ± 0.33	6.18 ± 0.08
	48	1.37 ± 0.15	3.72 ± 0.22	2.01 ± 0.12	1.09 ± 0.09
4T1	24	1.60 ± 0.14	2.20 ± 0.19	2.25 ± 0.34	2.37 ± 0.25
	48	1.62 ± 0.21	2.22 ± 0.25	1.94 ± 0.15	1.98 ± 0.21
HCC1954	24	2.52 ± 0.65	4.53 ± 0.18	9.91 ± 0.17	1.90 ± 0.11
	48	2.25 ± 0.64	5.01 ± 0.23	6.40 ± 0.25	1.15 ± 0.16
MCF-10A	24	93.01 ± 2.29	>100	>100	15.91 ± 0.91
	48	64.58 ± 0.68	>100	>100	0.23 ± 0.05

**Table 3:** The IC_50_ values of studied compounds in clonogenic assay.

Cell Line	IC_50_ (M ± SD)			
	Les-6287	Les-6294	Les-6328	Dox
MCF-7	0.42 ± 0.08	1.33 ± 0.10	1.57 ± 0.12	0.42 ± 0.08
MDA-MB-231	0.42 ± 0.11	0.67 ± 0.08	1.19 ± 0.03	0.42 ± 0.11
HCC1954	0.43 ± 0.11	3.05 ± 0.03	1.54 ± 0.02	0.43 ± 0.11
MCF-10A	>50	>50	>50	0.47 ± 0.11

**Table 4:** The IC_50_ values of studied compounds targeting cells in [^3^H]-thymidine assay.

Cell Line	IC_50_ (M ± SD)	
	Les-6287	Dox
MCF-7	2.37 ± 0.02	0.66 ± 0.13
MDA-MB-231	2.32 ± 0.04	0.63 ± 0.11
HCC1954	3.67 ± 0.09	1.07 ± 0.04
MCF-10A	43.54 ± 1.16	3.19 ± 0.43

## Data Availability

Data are contained within the article.

## References

[1] Sung H., Ferlay J., Siegel R.L., Laversanne M., Soerjomataram I., Jemal A., Bray F. (2021). Global Cancer Statistics 2020: GLOBOCAN Estimates of Incidence and Mortality Worldwide for 36 Cancers in 185 Countries. CA Cancer J. Clin..

[2] Philip C., Mathew A., John M.J. (2018). Cancer care: Challenges in the developing world. Cancer Res. Stat. Treat..

[3] Waks A.G., Winer E.P. (2019). Breast cancer treatment: A review. JAMA.

[4] Emran T.B., Shahriar A., Mahmud A.R., Rahman T., Abir M.H., Siddiquee M.F.-R., Ahmed H., Rahman N., Nainu F., Wahyudin E. (2022). Multidrug resistance in cancer: Understanding molecular mechanisms, immunoprevention and therapeutic approaches. Front. Oncol..

[5] Bass A.K.A., El-Zoghbi M.S., Nageeb E.-S.M., Mohamed M.F.A., Badr M., Abuo-Rahma G.E.-D.A. (2021). Abuo-Rahma, Comprehensive review for anticancer hybridized multitargeting HDAC inhibitors. Eur. J. Med. Chem..

[6] Xu Y., Gong M., Wang Y., Yang Y., Liu S., Zeng Q. (2023). Global trends and forecasts of breast cancer incidence and deaths. Sci. Data.

[7] Burguin A., Diorio C., Durocher F. (2021). Breast cancer treatments: Updates and new challenges. J. Pers. Med..

[8] Ronchi A., Pagliuca F., Zito Marino F., Accardo M., Cozzolino I., Franco R. (2021). Current and potential immunohistochemical biomarkers for prognosis and therapeutic stratification of breast carcinoma. Semin. Cancer Biol..

[9] Jacobs A.T., Martinez Castaneda-Cruz D., Rose M.M., Connelly L. (2022). Targeted therapy for breast cancer: An overview of drug classes and outcomes. Biochem. Pharmacol..

[10] Mark C., Lee J.S., Cui X., Yuan Y. (2023). Antibody-drug conjugates in breast cancer: Current status and future directions. Int. J. Mol. Sci..

[11] Nunes Filho P., Albuquerque C., Pilon Capella M., Debiasi M. (2023). Immune checkpoint inhibitors in breast cancer: A narrative review. Oncol. Ther..

[12] Swain S.M., Shastry M., Hamilton E. (2023). Targeting HER2-positive breast cancer: Advances and future directions. Nat. Rev. Drug Discov..

[13] Masoud V., Pagès G. (2017). Targeted therapies in breast cancer: New challenges to fight against resistance. World J. Clin. Oncol..

[14] Wang Y., Minden A. (2022). Current molecular combination therapies used for the treatment of breast cancer. Int. J. Mol. Sci..

[15] Yang T., Li W., Huang T., Zhou J. (2023). Antibody-drug conjugates for breast cancer treatment: Emerging agents, targets and future directions. Int. J. Mol. Sci..

[16] Campos J.C., Campos P.T., Bona N.P., Soares M.S., Souza P.O., Braganhol E., Cunico W., Siqueira G.M. (2022). Synthesis and biological evaluation of novel 2-imino-4-thiazolidinones as potential antitumor agents for glioblastoma. Med. Chem..

[17] Chawla P.A., Wahan S.K., Negi M., Faruk A., Chawla V. (2023). Synthetic strategies and medicinal perspectives of 4-thiazolidinones: Recent developments and structure–activity relationship studies. J. Heterocycl. Chem..

[18] Kadhim Z.Y., Alqaraghuli H.G.J., Abd M.T. (2021). Synthesis, characterization, molecular docking, in vitro biological evaluation and in vitro cytotoxicity Sstudy of novel thiazolidine-4-one derivatives as anti-breast cancer agents. Anti-Cancer Agents Med. Chem..

[19] Tahmasvand R., Bayat P., Vahdaniparast S.M., Dehghani S., Kooshafar Z., Khaleghi S., Almasirad A., Salimi M. (2020). Design and synthesis of novel 4-thiazolidinone derivatives with promising anti-breast cancer activity: Synthesis, characterization, in vitro and in vivo results. Bioorg. Chem..

[20] Subtel’na I., Atamanyuk D., Szymańska E., Kieć-Kononowicz K., Zimenkovsky B., Vasylenko O., Gzella A., Lesyk R. (2010). Synthesis of 5-arylidene-2-amino-4-azolones and evaluation of their anticancer activity. Bioorg. Med. Chem..

[21] Buzun K., Kryshchyshyn-Dylevych A., Senkiv J., Roman O., Gzella A., Bielawski K., Bielawska A., Lesyk R. (2021). Synthesis and anticancer activity evaluation of 5-[2-chloro-3-(4-nitrophenyl)-2-propenylidene]-4-thiazolidinones. Molecules.

[22] Buzun K., Gornowicz A., Lesyk R., Kryshchyshyn-Dylevych A., Gzella A., Czarnomysy R., Latacz G., Olejarz-Maciej A., Handzlik J., Bielawski K. (2022). 2-{5-[(Z,2Z)-2-Chloro-3-(4-nitrophenyl)-2-propenylidene]-4-oxo-2-thioxothiazolidin-3-yl}-3-methylbutanoic acid as a potential anti-breast cancer molecule. Int. J. Mol. Sci..

[23] Finiuk N., Kryshchyshyn-Dylevych A., Holota S., Klyuchivska O., Kozytskiy A., Karpenko O., Manko N., Ivasechko I., Stoika R., Lesyk R. (2022). Novel hybrid pyrrolidinedione-thiazolidinones as potential anticancer agents: Synthesis and biological evaluation. Eur. J. Med. Chem..

[24] Finiuk N., Kaleniuk E., Holota S., Stoika R., Lesyk R., Szychowski K.A. (2023). Pyrrolidinedione-thiazolidinone hybrid molecules with potent cytotoxic effect in squamous cell carcinoma SCC-15 cells. Bioorg. Med. Chem..

[25] Radomska D., Czarnomysy R., Szymanowska A., Radomski D., Domínguez-Álvarez E., Bielawska A., Bielawski K. (2022). Novel selenoesters as a potential tool in triple-negative breast cancer treatment. Cancers.

[26] Nunez J.G., Pinheiro J.S., Padilha G.L., Garcia H.O., Porta V., Apel M.A., Bruno A.N. (2020). Antineoplastic potential and chemical evaluation of essential oils from leaves and flowers of Tagetes ostenii Hicken. An. Acad. Bras. Cienc..

[27] Szwed A., Miłowska K., Michlewska S., Moreno S., Shcharbin D., Gomez-Ramirez R., de la Mata F.J., Majoral J.-P., Bryszewska M., Gabryelak T. (2020). Generation dependent effects and entrance to mitochondria of hybrid dendrimers on normal and cancer neuronal cells in vitro. Biomolecules.

[28] Avrutsky M.I., Troy C.M. (2021). Caspase-9: A multimodal therapeutic target with diverse cellular expression in human disease. Front. Pharmacol..

[29] Gornowicz A., Lesyk R., Czarnomysy R., Holota S., Shepeta Y., Popławska B., Podolak M., Szymanowski W., Bielawski K., Bielawska A. (2023). Multi-targeting anticancer activity of a new 4-thiazolidinone derivative with anti-HER2 antibodies in human AGS gastric cancer cells. Int. J. Mol. Sci..

[30] Zhang S., Rao S., Yang M., Ma C., Hong F., Yang S. (2022). Role of mitochondrial pathways in cell apoptosis during hepatic ischemia/reperfusion injury. Int. J. Mol. Sci..

[31] Jiang M., Qi L., Li L., Wu Y., Song D., Li Y. (2021). Caspase-8: A key protein of cross-talk signal way in “panoptosis” in cancer. Int. J. Cancer.

[32] Singh V., Khurana A., Navik U., Allawadhi P., Bharani K.K., Weiskirchen R. (2022). Apoptosis and pharmacological therapies for targeting thereof for cancer therapeutics. Science.

[33] Talvensaari-Mattila A., Pääkkö P., Turpeenniemi-Hujanen T. (2003). Matrix metalloproteinase-2 (MMP-2) is associated with survival in breast carcinoma. Br. J. Cancer.

[34] Huang H. (2018). Matrix metalloproteinase-9 (MMP-9) as a cancer biomarker and MMP-9 biosensors: Recent advances. Sensors.

[35] Quintero-Fabián S., Arreola R., Becerril-Villanueva E., Torres-Romero J.C., Arana-Argáez V., Lara-Riegos J., Ramírez-Camacho M.A., Alvarez-Sánchez M.E. (2019). Role of matrix metalloproteinases in angiogenesis and cancer. Front. Oncol..

[36] Hossain M., Habib I., Singha K., Kumar A. (2024). FDA-approved heterocyclic molecules for cancer treatment: Synthesis, dosage, mechanism of action and their adverse effect. Heliyon.

[37] Singh A.K., Kumar A., Singh H., Sonawane P., Paliwal H., Thareja S., Pathak P., Grishina M., Jaremko M., Emwas A.-H. (2022). Concept of hybrid drugs and recent advancements in anticancer hybrids. Pharmaceuticals.

[38] Poyraz S., Döndaş H.A., Döndaş N.Y., Sansano J.M. (2023). Recent insights about pyrrolidine core skeletons in pharmacology. Front. Pharmacol..

[39] Wang Y., Wang N., Chen Y., Yang Y. (2023). Regulation of micropatterned curvature-dependent FA heterogeneity on cytoskeleton tension and nuclear DNA synthesis of malignant breast cancer cells. J. Mater. Chem. B.

[40] Shadbad M.A., Safaei S., Brunetti O., Derakhshani A., Lotfinejad P., Mokhtarzadeh A., Hemmat N., Racanelli V., Solimando A.G., Argentiero A. (2021). A systematic review on the therapeutic potentiality of PD-L1-inhibiting microRNAs for triple-negative breast cancer: Toward single-cell sequencing-guided biomimetic delivery. Genes.

[41] Yuan J., Ofengeim D. (2023). A guide to cell death pathways. Nat. Rev. Mol. Cell Biol..

[42] Van Opdenbosch N., Lamkanfi M. (2019). Caspases in cell death, inflammation, and disease. Immunity.

[43] Wang S., He M., Li L., Liang Z., Zou Z., Tao A. (2016). Cell-in-cell death is not restricted by caspase-3 deficiency in MCF-7 cells. J. Breast Cancer.

[44] Onorati A.V., Dyczynski M., Ojha R., Amaravadi R.K. (2018). Targeting autophagy in cancer. Cancer.

[45] Wirawan E., Vande Walle L., Kersse K., Cornelis S., Claerhout S., Vanoverberghe I., Roelandt R., De Rycke R., Verspurten J., Declercq W. (2010). Caspase-mediated cleavage of Beclin-1 inactivates Beclin-1-induced autophagy and enhances apoptosis by promoting the release of proapoptotic factors from mitochondria. Cell Death Dis..

[46] Jiang H., Li H. (2021). Prognostic values of tumoral MMP2 and MMP9 overexpression in breast cancer: A systematic review and meta-analysis. BMC Cancer.

[47] Dong H., Diao H., Zhao Y., Xu H., Pei S., Gao J., Wang J., Hussain T., Zhao D., Zhou X. (2019). Overexpression of matrix metalloproteinase-9 in breast cancer cell lines remarkably increases the cell malignancy largely via activation of transforming growth factor beta/SMAD signalling. Cell Prolif..

[48] Chen M., Wu C., Fu Z., Liu S. (2022). ICAM1 promotes bone metastasis via integrin-mediated TGF-β/EMT signaling in triple-negative breast cancer. Cancer Sci..

